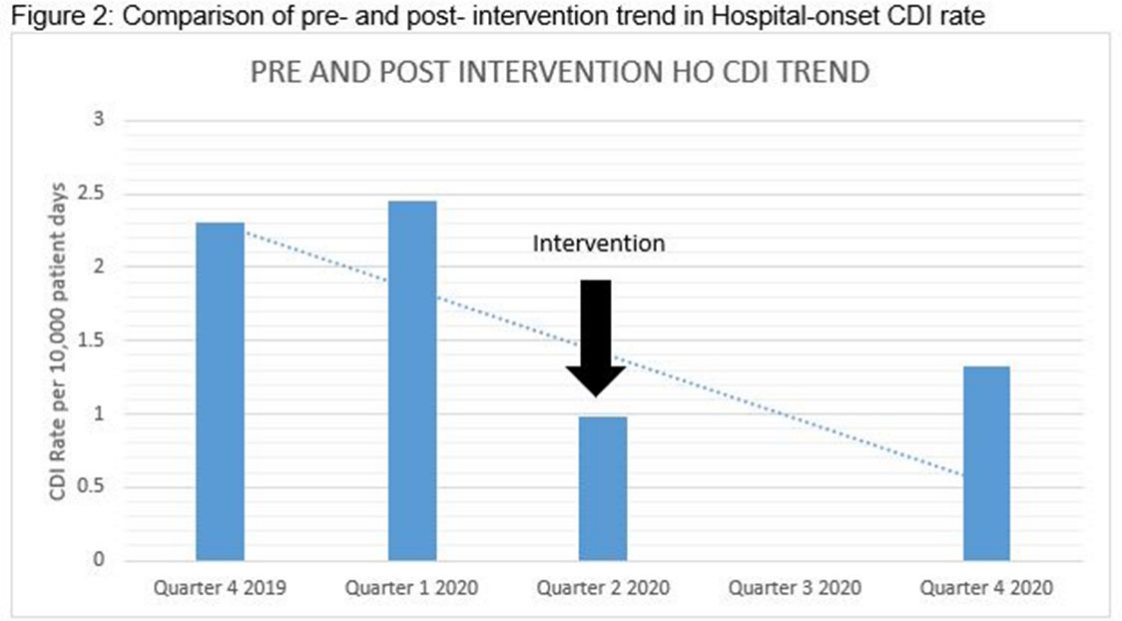# Impact of Two-Step Testing Algorithm on Reducing Hospital-Onset *Clostridioides difficile* Infections

**DOI:** 10.1017/ash.2021.78

**Published:** 2021-07-29

**Authors:** Bhagyashri Navalkele, Wendy Winn, Sheila Fletcher, Regina Galloway, Jason Parham, William Daley, Patrick Kyle, Vonda Clack, Kathy Sheilds

## Abstract

*Clostridioides difficile* infection (CDI) is one of the leading causes of hospital–onset infections. Clinically distinguishing true CDI versus colonization with *C. difficile* is challenging and often requires reliable and rapid molecular testing methods. At our academic center, we implemented a 2-step testing algorithm to help identify true CDI cases. The University of Mississippi Medical Center is a 700+ bed academic facility located in Jackson, Mississippi. Hospital-onset (HO) CDI was defined based on NHSN Laboratory Identified (LabID) event as the last positive *C. difficile* test result performed on a specimen using a multistep testing algorithm collected >3 calendar days after admission to the facility. HO-CDI data were collected from all inpatient units except the NICU and newborn nursery. HO-CDI outcomes were assessed based on standardized infection ratio (SIR) data. In May 2020, we implemented a 2-step testing algorithm (Figure 1). All patients with diarrhea underwent *C. difficile* PCR testing. Those with positive *C. difficile* PCR test were reflexed to undergo enzyme immunoassay (EIA) glutamate dehydrogenase antigen (Ag) testing and toxin A and B testing. The final results were reported as colonization (*C. difficile* PCR+/EIA Ag+/Toxin A/B−) or true CDI case (*C. difficile* PCR+/EIA +/Toxin A/B +) or negative (*C. difficile* PCR−). All patients with colonization or true infection were placed under contact isolation precautions until diarrhea resolution for 48 hours. During the preintervention period (October 2019–April 2020), 25 HO-CDI cases were reported compared to 8 cases in the postintervention period (June 2020–December 2020). A reduction in CDI SIR occurred in the postintervention period (Q3 2020–Q4 2020, SIR 0.265) compared to preintervention period (Q4 2019–Q1 2020, SIR 0.338) (Figure 2). We successfully reduced our NHSN HO-CDI SIR below the national average after implementing a 2-step testing algorithm for CDI. The 2-step testing algorithm was useful for antimicrobial stewardship to guide appropriate CDI treatment for true cases and for infection prevention to continue isolation of infected and colonized cases to reduce the spread of *C. difficile* spores.

**Funding:** No

**Disclosures:** None

**Figure 1.**
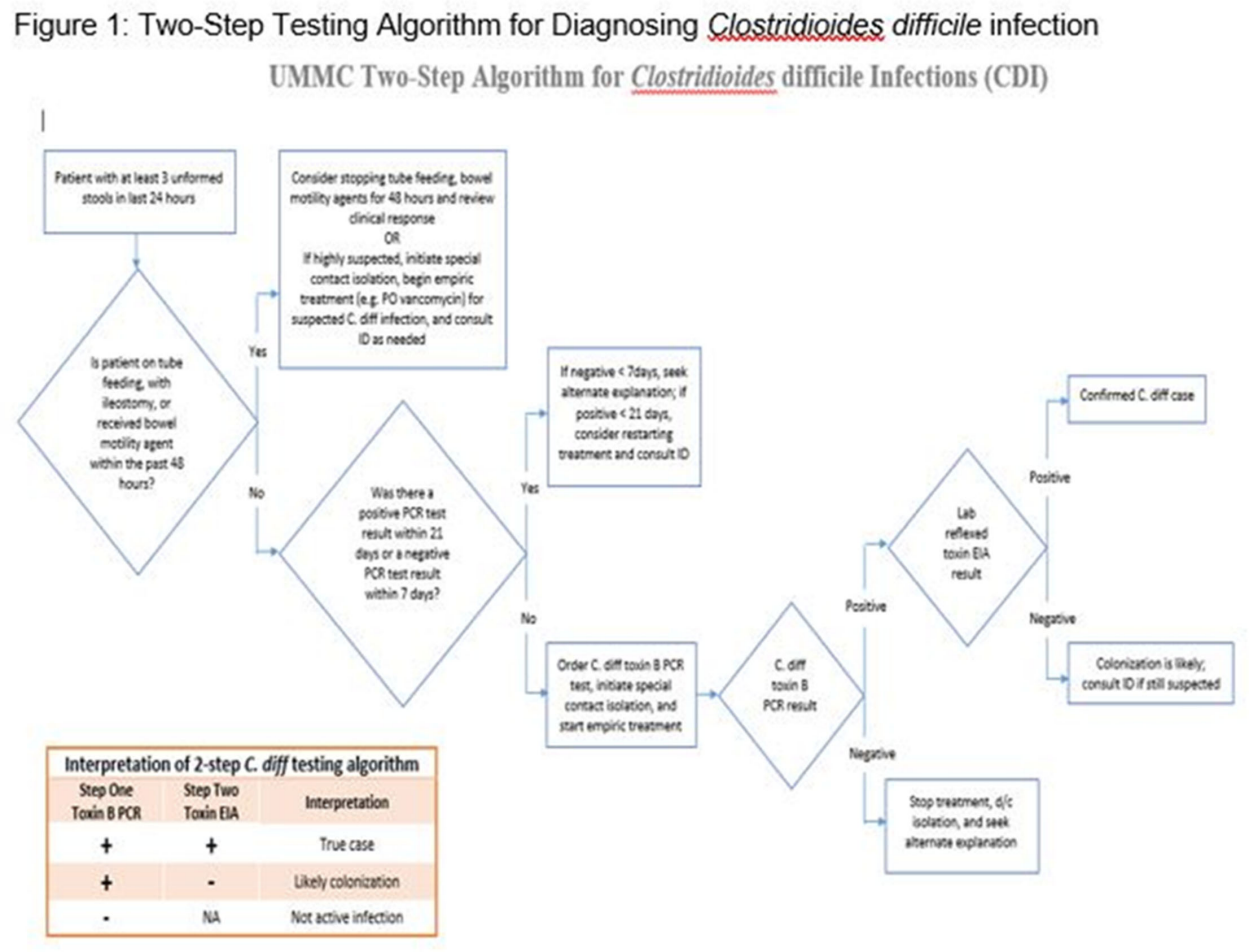

**Figure 2.**